# The Central Role of Neuronal Cell Death in Alzheimer’s Disease Pathobiology

**DOI:** 10.3390/biomedicines14050953

**Published:** 2026-04-22

**Authors:** Soyoung Kwak, Jin Kyung Kim, Yong-Uk Lee, Hye Suk Baek, Ye Jin Kwon, Mee-Na Park, Jeong-Ho Hong, Seung-Bo Lee, Hae Won Kim, Shin Kim

**Affiliations:** 1 Department of Nuclear Medicine, Keimyung University Dongsan Hospital, Daegu 42601, Republic of Korea; ksy845@gmail.com (S.Y.K.); kyjj0214@naver.com (Y.J.K.); 2 Department of Microbiology, Keimyung University Dongsan Hospital, Daegu 42601, Republic of Korea; pcjlovesh6@dsmc.or.kr (J.K.K.); yulee@dsmc.or.kr (Y.-U.L.); 3 Department of Immunology, School of Medicine, Keimyung University, Daegu 42601, Republic of Korea; sftwtmt@hanmail.net (H.S.B.); parkmn1223@gmail.com (M.-N.P.); 4 Department of Neurology, Keimyung University Dongsan Hospital, Daegu 42601, Republic of Korea; neurohong79@gmail.com; 5 Department of Medical Informatics, School of Medicine, Keimyung University, Daegu 42601, Republic of Korea; koreateam23@gmail.com

**Keywords:** Alzheimer’s disease, regulated cell death, apoptosis, necroptosis, pyroptosis, ferroptosis, autophagy–lysosome dysfunction, biomarkers

## Abstract

Alzheimer’s disease (AD) is a progressive neurodegenerative disorder in which amyloid β accumulation, tau pathology, chronic neuroinflammation, and metabolic stress converge to drive synaptic dysfunction and neuronal loss. Rather than resulting from a single mechanism, increasing evidence indicates that neurodegeneration in AD is mediated by the coordinated activation of multiple regulated cell death pathways. These pathways include apoptosis, necroptosis, pyroptosis, ferroptosis, and autophagy-dependent cell death, each characterized by distinct molecular mediators and execution programs. Evidence from human brain tissues, animal models, and in vitro systems demonstrates that core pathological drivers such as amyloid β and tau pathology, oxidative stress, and sustained neuroinflammation engage these death pathways in a spatially, temporally, and cell-type-dependent manner across neurons and glial populations. In this review, we synthesize the current knowledge on regulated cell death mechanisms in AD, emphasizing their molecular signatures, cellular specificity, and stage-dependent involvement, together with recent advances in immunohistochemical, imaging, and biofluid-based approaches for detecting neuronal death. By integrating evidence across molecular, cellular, and system levels, this review positions regulated cell death as a unifying framework for understanding neurodegeneration and developing pathway-specific biomarkers and combinatorial neuroprotective strategies.

## 1. Introduction

Alzheimer’s disease (AD) is a progressive neurodegenerative disorder marked by synaptic dysfunction and neuronal loss, leading to cognitive decline and dementia [1]. Although amyloid β (Aβ) deposition and tau aggregation are central pathological features of AD, it develops through the interplay of multiple processes, including chronic neuroinflammation, oxidative and metabolic stress, mitochondrial dysfunction, impaired proteostasis, and vascular and immune dysregulation. These factors collectively impose sustained stress on neurons and glial cells, heightening their vulnerability to degeneration [2]. Neuronal loss defines AD progression, making regulated cell death (RCD) a critical determinant of disease outcome [3]. Evidence indicates that neuronal loss in AD is accompanied by signatures of multiple RCD mechanisms, including apoptosis, necroptosis, pyroptosis, ferroptosis, and autophagy-dependent cell death. These pathways are driven by distinct molecular machinery, and may act in parallel or sequentially, amplifying neurodegeneration [4,5,6,7,8]. Within the current framework of AD pathogenesis, the amyloid β and tau pathologies are generally regarded as upstream triggers, whereas RCD pathways represent downstream but actively regulated processes that translate pathological stress into neuronal injury. Importantly, accumulating evidence suggests that several RCD-associated mechanisms are not merely terminal consequences but actively contribute to disease progression by exacerbating core pathological features. This is supported by multiple mechanistic observations linking specific RCD pathways to core pathological processes in AD. Inflammasome activation represents a central component of pyroptotic signaling and contributes to amyloid aggregation and tau pathology [9,10]. Iron dysregulation associated with ferroptotic stress is closely linked to amyloid accumulation and oxidative injury [11,12], and impaired autophagy facilitates intracellular Aβ accumulation [13,14,15].

These observations support a model in which RCD pathways function as both effectors and amplifiers of neurodegeneration, linking upstream pathological triggers to progressive neuronal loss while simultaneously reinforcing disease-driving mechanisms. Thus, rather than serving solely as passive endpoints, RCD-related processes may actively modulate the trajectory of AD progression through interconnected metabolic, inflammatory, and proteostatic feedback networks.

This review outlines the major RCD pathways implicated in AD, including apoptosis, necroptosis, pyroptosis, ferroptosis, and autophagy-dependent cell death. By integrating findings from human neuropathology, experimental models, and in vitro systems, this review aims to (i) delineate the molecular signatures and cellular specificity of each pathway, (ii) highlight their stage-dependent involvement in disease progression, (iii) evaluate emerging biomarkers that capture pathway activity, and (iv) discuss the implications of RCD integration for therapeutic development. This review positions RCD as a unifying framework for understanding neurodegeneration in AD and guiding the design of biomarker-driven neuroprotective strategies.

**Figure 1:**
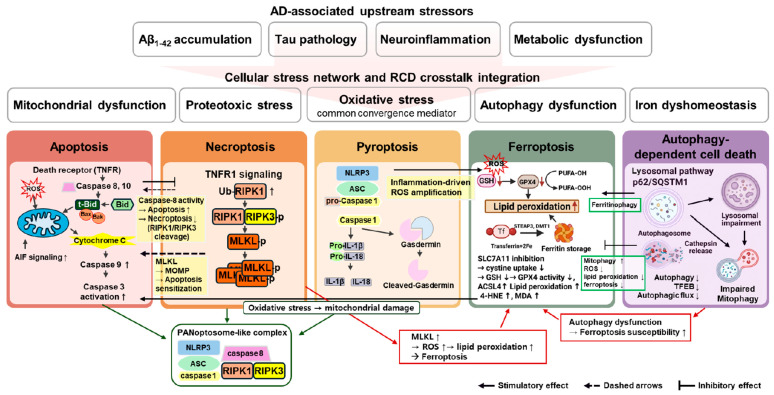
Integrated network of upstream pathological drivers and regulated cell death pathways in Alzheimer’s disease. Key AD-associated factors, including amyloid β (Aβ), tau pathology, and neuroinflammation, induce shared cellular stress responses such as oxidative stress, mitochondrial dysfunction, proteotoxic stress, iron dyshomeostasis, and lysosomal/autophagy impairment. These stressors collectively promote multiple regulated cell death (RCD) pathways. Apoptosis is mediated via mitochondrial and death receptor signaling. Necroptosis involves RIPK1–RIPK3 signaling and MLKL phosphorylation, contributing to membrane disruption. Pyroptosis is driven by inflammasome activation, leading to caspase-1 activation, gasdermin D (GSDMD) cleavage, and pro-inflammatory cytokine release. Ferroptosis is associated with iron-dependent lipid peroxidation and impaired glutathione peroxidase 4 (GPX4) activity. Autophagy-dependent cell death is linked to defective autophagic flux and lysosomal dysfunction. These pathways are not independent but are coordinated within an interconnected regulatory network, in which shared stress signals and molecular checkpoints modulate pathway crosstalk and influence cell death outcomes in a context-dependent manner. Aβ, amyloid β; AD, Alzheimer’s disease; RCD, regulated cell death; ROS, reactive oxygen species; RIPK, receptor-interacting protein kinase; MLKL, mixed lineage kinase domain-like protein; p-MLKL, phosphorylated MLKL; TNFR, tumor necrosis factor receptor; NLRP3, NOD-like receptor family pyrin domain containing 3; ASC, apoptosis-associated speck-like protein containing a CARD; GSDMD, gasdermin D; Bid, BH3-interacting domain death agonist; tBid, truncated Bid; AIF, apoptosis-inducing factor; Caspase-1, cysteine-aspartic protease-1; Caspase-3, cysteine-aspartic protease-3; Caspase-8, cysteine-aspartic protease-8; BAX, Bcl-2-associated X protein; BAK, Bcl-2 antagonist/killer; MOMP, mitochondrial outer membrane permeabilization; GPX4, glutathione peroxidase 4; GSH, glutathione; PUFA, polyunsaturated fatty acid; ACSL4, acyl-CoA synthetase long-chain family member 4; STEAP3, six-transmembrane epithelial antigen of prostate 3; SLC7A11, solute carrier family 7 member 11 (xCT); Fe^2+^, ferrous iron; 4-HNE, 4-hydroxynonenal; MDA, malondialdehyde; IL-1β, interleukin-1 beta; IL-18, interleukin-18; TFEB, transcription factor EB.

Accordingly, this review positions RCD not as a primary initiating cause of AD, but as mechanistically informative and clinically actionable processes that both reflect and influence disease progression, with relevance for biomarker development and therapeutic intervention. The following sections examine each cell death modality in detail, highlighting evidence of activation in AD, and implications for diagnosis and therapeutic development. To provide a conceptual overview of this integrated framework, Figure 1 illustrates the convergence of upstream pathological drivers and coordinated engagement of multiple RCD pathways in AD.

## 2. Apoptosis in AD

Apoptosis is a regulated form of programmed cell death characterized by cell shrinkage, chromatin condensation, DNA fragmentation, and caspase activation [16]. It can be initiated through extrinsic death receptor signaling or intrinsic mitochondrial pathways, both converging on executioner caspases that dismantle the cell [17,18]. In AD, apoptosis has long been studied as a potential contributor to neuronal loss. Yet, its prevalence and significance remain debated, with evidence suggesting that apoptotic signaling may occur in a context- or stage-specific manner rather than as the dominant mode of neuronal death.

### 2.1. Neuropathological and Experimental Evidence for Apoptotic Neurodegeneration in AD

Postmortem analyses of AD brains have revealed extensive apoptosis-associated nuclear damage in vulnerable cortical and hippocampal regions. Notably, apoptosis-associated pathology is detected not only in neurons but also in astrocytes and microglia, suggesting that neurodegeneration in AD involves multicellular responses beyond isolated neuronal loss [19,20,21]. These findings align with evidence of a pro-death intracellular milieu characterized by the dysregulation of mitochondrial death pathways and an imbalance between pro- and anti-survival signaling components [22,23]. Apoptotic signaling is frequently observed in neurons proximal to amyloid plaques and neurofibrillary tangles, suggesting that Aβ and tau pathologies are closely linked to mitochondrial dysfunction, which may increase neuronal vulnerability to degeneration rather than acting as direct and immediate triggers of cell death [23,24,25,26]. Consistent with this, tau pathology contributes to apoptotic signaling through mitochondrial dysfunction and activation of intrinsic caspase pathways, as hyperphosphorylated tau disrupts mitochondrial integrity and promotes cytochrome c release, thereby facilitating caspase-dependent neuronal apoptosis [27].

Despite strong molecular signatures, classical apoptotic hallmarks such as chromatin condensation and nuclear fragmentation are inconsistently observed in postmortem AD brains. Rather, many neurons exhibit necroptotic-like or atypical degenerative morphologies. This apparent discrepancy may reflect differences in disease stage or cellular context, where neurons exhibit features of multiple death-related processes prior to irreversible degeneration. The coexistence of molecular apoptotic markers and heterogeneous morphological features suggests that neuronal death in AD may involve overlapping and non-exclusive cell death pathways, rather than a single discrete mechanism [28,29].

Experimental models further support apoptosis as a downstream effector of neurodegenerative processes in AD, reflecting cellular responses to upstream pathological stressors, such as amyloid and tau accumulation. In primary neuronal cultures, Aβ1-42 exposure induces mitochondrial dysfunction, intrinsic death signaling, and caspase-dependent apoptosis, directly linking amyloid burden to apoptotic engagement [30]. Early apoptotic signaling may occur locally within synapses and dendrites, preceding full neuronal apoptosis and contributing to the slow progression of neurodegeneration [31]. Beyond neuron-autonomous toxicity, Aβ1-42 also activates innate immune receptors such as Toll-like receptor 4 (TLR4) in microglia, triggering inflammatory signaling cascades that amplify apoptotic stress in neighboring neurons. Specifically, TLR4-mediated activation of the NF-κB and MAPK/JNK pathways promotes the release of pro-inflammatory cytokines and reactive oxygen species, which converge on mitochondrial dysfunction and caspase activation [32]. Consistently, TLR4 knockdown has been reported to attenuate inflammatory responses and reduce caspase-associated apoptotic readouts in AD-relevant cellular models, implicating receptor-mediated, non-cell-autonomous pathways in Aβ-driven apoptotic injury.

Metabolic and stress-response modulators also influence apoptotic susceptibility in AD models. In neuronal systems, mitochondrial sirtuin SIRT4 is upregulated under AD-related conditions and enhances neuronal apoptosis by exacerbating mitochondrial dysfunction and caspase activation in the presence of Aβ pathology, thereby linking metabolic dysregulation to intrinsic apoptotic signaling [33]. Similarly, in human SH-SY5Y neuroblastoma cells, combined exposure to lipopolysaccharide and Aβ1-42 upregulates pro-apoptotic genes, including BAX and caspase-3, increases caspase-dependent apoptotic cell death, and reduces cell viability, providing direct experimental evidence that inflammatory and amyloid-associated stressors synergistically promote apoptosis [34]. Pharmacological intervention with hesperetin partially rescues these effects by attenuating stress-associated MAPK/JNK signaling and downstream caspase activation, thereby suppressing apoptotic marker expression [35,36]. These findings highlight the therapeutic potential of targeting apoptosis under AD-related stress conditions.

In vivo evidence from the 5xFAD mouse model confirms that amyloid pathology is accompanied by measurable neuronal apoptosis. Symptomatic 5xFAD mice show cleaved caspase-3 immunoreactivity alongside established neurodegeneration and neuronal loss markers, including Fluoro-Jade C and Nissl staining, confirming the presence of apoptosis at the tissue level [37]. 5xFAD brains exhibit increased activation of the pro-apoptotic Bcl-2 family regulator BAD, identifying BAD-dependent signaling as a contributor to neuronal apoptosis and associated neuroinflammatory responses in this model [38].

Genetic suppression of BAD in 5xFAD mice significantly reduces neuronal caspase-3 activation, attenuates neuroinflammation and neuronal degeneration in vulnerable brain regions, and partially restores learning and memory, demonstrating that apoptosis actively contributes to disease progression as a dynamically regulated downstream effector that amplifies neuronal vulnerability in response to amyloid-associated pathology [38]. Consistent with this interpretation, recent studies have further demonstrated that dysregulation of mitochondrial homeostasis regulators (such as MST1 or DHCR24) in 5xFAD mice also increases apoptotic markers, including Bax/Bcl-2 imbalance and caspase activation, whereas modulation of these pathways reduces apoptosis and improves neuronal survival and cognition [39,40]. Thus, in 5xFAD brains, apoptosis is experimentally detectable, regionally patterned, and mechanistically linked to neuronal degeneration.

Collectively, these neuropathological and experimental findings establish apoptosis as a mechanistically relevant downstream component of AD-related neurodegeneration. Nevertheless, apoptosis alone cannot explain the full extent and heterogeneity of neuronal loss observed in AD, indicating that it functions within a broader, multi-modal cell death landscape.

### 2.2. Molecular and Cellular Biomarkers of Apoptosis in AD

The experimental detection of apoptosis in AD relies on a range of biomarkers that reflect distinct stages within the apoptotic cascade, from early signaling events to terminal execution [4,19,30,41] (Table 1). At the nuclear level, DNA fragmentation is a hallmark of late-stage apoptosis, and in situ labeling assays such as TUNEL are widely used as tissue-based markers for identifying apoptosis-associated DNA damage in AD cortical and hippocampal specimens.

At the enzymatic level, caspase activation is central to apoptotic progression. Caspase-8, an initiator of extrinsic death receptor signaling, has been implicated in AD pathology and interacts with downstream effectors to influence both apoptotic and alternative cell death pathways. During this process, BH3-interacting domain death agonist (Bid) cleavage by caspase-8 generates truncated Bid (t-Bid), which links death receptor signaling to mitochondrial apoptotic activation. Caspase-9, activated after mitochondrial membrane permeabilization, drives intrinsic signaling, whereas caspase-3, the main executioner protease, cleaves structural proteins and DNA fragmentation factors. In AD-related stress models, combined Aβ and inflammatory stimuli upregulate caspase-3, caspase-8, and caspase-9, contributing to neuronal injury and death. These dynamics have been demonstrated in human neuronal cell models exposed to Aβ1-42 in vitro.

At the mitochondrial level, dysregulation of the Bcl-2 family proteins represents a core apoptotic biomarker axis in AD. An increased Bax/Bcl-2 ratio reflects mitochondrial membrane permeabilization and susceptibility to intrinsic apoptotic signaling [42]. Additional Bcl-2 family proteins, including BAD, further modulate this balance by promoting pro-apoptotic signaling [38]. These mitochondrial biomarkers are consistently observed across experimental models and correlate with neuronal vulnerability and degeneration [42]. Additionally, regulators of mitochondrial homeostasis, including MST1 and DHCR24, have emerged as upstream modulators of apoptotic susceptibility. Rather than serving as direct execution markers, these factors influence mitochondrial integrity and cellular stress adaptation, thereby lowering the threshold for mitochondrial membrane permeabilization and apoptotic commitment [39,40]. Importantly, apoptosis-associated biomarkers are dynamically responsive to therapeutic interventions. Changes in execution-phase markers, particularly cleaved caspase-3, have been consistently associated with improved neuronal survival and functional outcomes, highlighting their potential as translational biomarkers for monitoring treatment efficacy in AD [45,46,47].

In addition to caspase-dependent mechanisms, caspase-independent pathways contribute to apoptotic signaling. Apoptosis-inducing factor (AIF) translocation from the mitochondria to the nucleus indicates the activation of caspase-independent apoptotic pathways, leading to large-scale DNA fragmentation and chromatin condensation independent of caspase activity. Elevated AIF nuclear immunoreactivity has been reported in AD hippocampi compared with controls, consistent with non-caspase-dependent apoptotic mechanisms [43]. In parallel, transcriptional regulators contribute to apoptotic modulation. Transcriptional regulators, such as activator protein-1 components c-Jun and c-Fos, often activated downstream of stress kinases, including JNK, function upstream of mitochondrial and caspase-dependent pathways, linking cellular stress responses to apoptotic signaling cascades and aligning with pro-apoptotic signaling changes in degenerating neurons [44]. An altered proBDNF/BDNF balance further biases neurotrophin signaling toward p75 neurotrophin receptor-dependent MAPK/JNK activation, leading to downstream caspase-dependent apoptosis [48,49]. While the above biomarkers are primarily characterized in tissue-based systems, recent studies have expanded their detection to biofluids. In cerebrospinal fluid (CSF), apoptosis-related signals are increasingly detected through neuron-associated biomolecules, including cell-free DNA fragments and neuron-derived extracellular vesicles carrying apoptosis-related proteins. These markers provide a minimally invasive platform for assessing neuronal injury and cell death processes in AD [50,51]. In addition, alterations in cell-free mitochondrial DNA levels in CSF are thought to reflect early mitochondrial dysfunction rather than direct apoptotic cell death, providing an upstream indicator of apoptotic susceptibility in AD [51,52]. Although these circulating biomarkers are associated with apoptosis, they are not exclusively specific and may also reflect overlapping RCD pathways, including necroptosis and other stress-related processes.

Collectively, nuclear, enzymatic, mitochondrial, and transcriptional biomarkers including caspase activity, Bcl-2 family imbalance, mitochondrial stress indicators, and stress-responsive transcriptional and receptor-linked signals enable multi-level characterization of apoptosis in AD across human tissue, experimental models, and in vitro systems. Integrated with findings on caspase activation dynamics, mitochondrial susceptibility, and upstream stress-response pathways, these biomarker signatures support the concept that apoptosis represents a dynamically regulated and mechanistically integrated component of AD pathology, interacting with other forms of RCD and contributing to disease progression in a temporally staged manner.

## 3. Necroptosis in AD

Necroptosis is a caspase-independent, kinase-driven form of RCD characterized by the assembly of the receptor-interacting protein kinase-1 (RIPK1)–RIPK3 signaling complex and subsequent phosphorylation of mixed lineage kinase domain-like protein (MLKL), which subsequently oligomerizes and can translocate to the plasma membrane, where it is associated with membrane permeabilization and lytic cell death, representing a critical molecular factor in necroptotic signaling. This process releases intracellular damage-associated molecular patterns, making necroptosis intrinsically pro-inflammatory and tightly linked to sustained immune activation. In AD, postmortem neuropathological analyses have demonstrated the selective activation of RIPK1, RIPK3, and phosphorylated MLKL in the hippocampal and cortical regions most affected by pathology [53]. The regional abundance of these markers parallels the indices of microglial activation and inflammatory cytokine signaling, supporting a mechanistic link between chronic neuroinflammation and RIPK1–RIPK3–MLKL-dependent necroptotic signaling in vulnerable neuronal populations [54].

### 3.1. Neuropathological and Experimental Evidence for Necroptotic Neurodegeneration in AD

Necroptosis has emerged as an increasingly supported contributor to neuronal loss in AD. Postmortem studies in AD have demonstrated increased activation of necroptotic signaling components, including RIPK1, RIPK3, and phosphorylated MLKL, in vulnerable cortical and hippocampal regions [54]. These alterations occur in parallel with increased microglial activation and inflammatory cytokine signaling reported in human AD brains [55], supporting an association between chronic neuroinflammation and necroptotic pathway engagement in affected neuronal populations, as further summarized in recent systematic reviews [56] (Table 2).

Quantitative neuropathological analyses have shown that activation of the canonical necroptosis machinery, RIPK1, RIPK3, and phosphorylated MLKL (p-MLKL), scales with AD severity and is mechanistically linked to tau-associated neurodegeneration [58]. In the human AD hippocampus, p-MLKL immunoreactivity is not diffusely distributed but is selectively enriched within granulovacuolar degeneration (GVD) lesions, a hallmark of vulnerable neurons. The burden of p-MLKL-positive GVD neurons correlates with the Braak neurofibrillary tangle stage and neuronal loss [59], supporting GVD as a reproducible morphological correlate of necroptotic vulnerability rather than a nonspecific stress marker [60]. Together, these findings suggest that multiple proteinopathies, including tau, converge on necroptotic signaling, where tau pathology may further amplify the associated inflammatory and degenerative processes in AD pathophysiology [58].

This association is strengthened in cases of co-pathology. In AD with concomitant TDP-43 pathology, classified as limbic-predominant age-related TDP-43 encephalopathy, GVD-associated p-MLKL pathology is significantly increased. Phosphorylated TDP-43 is detected within subsets of p-MLKL-positive granules, indicating the convergence of multiple proteinopathy-driven stress processes on the same hippocampal neuronal pathway characterized by GVD-linked necroptotic activation [60].

Necroptotic signaling is also engaged early in AD. In individuals with mild cognitive impairment, considered prodromal AD, postmortem and transcriptomic evidence shows increased necroptosis component expression compared with controls, alongside suppression of neuroprotective pathways such as reduced expression of the transcriptional co-activator YAP (Yes-associated protein) in vulnerable hippocampal neurons [63]. These findings suggest that early disruption of YAP-mediated neuroprotection permits premature necroptotic engagement, contributing to synaptic dysfunction and neuronal vulnerability before extensive amyloid or tau accumulation.

Experimental models have provided causal evidence for necroptosis in AD-related neurodegeneration. In AD models, activation of RIPK1–RIPK3–MLKL signaling is closely associated with neuronal loss and neuroinflammatory responses, whereas inhibition of this pathway has been shown to attenuate neurodegeneration and preserve neuronal integrity, supporting a contributory role of necroptotic signaling in disease progression. The transition from pathway activation to irreversible cell death is likely governed by context-dependent regulatory mechanisms [54,58]. At the cellular level, RIPK1/RIPK3-dependent necroptosis is tightly coupled to inflammatory signaling cascades, further amplifying microglial activation and neuronal damage. These findings collectively indicate that necroptotic signaling is not merely a passive byproduct of neurodegeneration but a regulated downstream pathway that modulates inflammatory and degenerative responses following upstream pathological processes.

Collectively, these human and experimental findings establish necroptosis as an active, inflammation-associated mechanism of neuronal loss in AD that can be engaged early and causally modulated rather than as a purely downstream consequence of cumulative neuronal injury, underscoring its potential for therapeutic intervention across the continuum of AD progression.

### 3.2. Molecular and Cellular Biomarkers of Necroptosis in AD

Accurate identification of necroptosis in AD requires biomarkers that distinguish regulated necroptosis from apoptosis and other death pathways. The RIPK1–RIPK3–MLKL axis defines necroptosis and provides a hierarchical set of biomarkers for its initiation, execution, and suppression [64].

At the molecular level, increased expression and phosphorylation of RIPK1 and RIPK3 indicate upstream pathway engagement. Quantitative analyses of human AD hippocampus show elevated RIPK1 and RIPK3 in vulnerable regions, particularly under heightened neuroinflammation, supporting their use as histopathological biomarkers of necroptotic activation rather than nonspecific injury [58]. Cell-type-resolved studies have revealed the differential localization of RIPK1 and RIPK3 in neurons and glia, reflecting their dual roles in inflammatory integration and death execution, thereby enhancing their interpretive value as context-dependent biomarkers [61].

Downstream of pathway initiation, phosphorylation, and membrane translocation of MLKL represent the terminal and most specific molecular hallmark of necroptosis. p-MLKL is consistently detected in vulnerable neuronal populations in human AD tissue, enriched within granulovacuolar degeneration (GVD) structures in the hippocampus. Its burden correlates with Braak stage and neuronal loss [58,65]. Because p-MLKL directly mediates membrane disruption, it is widely regarded as a key marker of necroptotic signaling activation. However, the detection of p-MLKL, is best interpreted within a broader molecular context, as it may reflect regulated or intermediate signaling states rather than terminal cell death [58]. The discrimination between necroptosis and apoptosis relies on the combined assessment of RIPK signaling and caspase activity. Caspase-8 suppresses necroptosis by cleaving RIPK1 and RIPK3. Thus, elevated RIPK3 and p-MLKL levels in the absence of caspase-3 or caspase-8 activation strongly indicate necroptotic rather than apoptotic execution [60,61]. Evaluating this reciprocal balance provides a robust framework for stratifying cell death modalities in AD tissues.

Recent translational advances have extended the assessment of necroptosis-related biomarkers beyond post-mortem tissues through the development of RIPK1-targeted molecular imaging approaches. Preclinical studies using 11C/18F-labeled RIPK1 inhibitors have demonstrated the feasibility of in vivo PET imaging of RIPK1 expression in the brain, supporting their potential utility in evaluating neuroinflammation and necroptotic signaling in neurodegenerative diseases [57]. These approaches highlight RIPK1-based PET imaging as a promising noninvasive strategy for assessing inflammation-associated necroptotic signaling in vivo [57,66].

Taken together, combined assessment of RIPK1/RIPK3 signaling, p-MLKL, caspase activity, and emerging RIPK1-targeted PET imaging may improve the identification of necroptosis in AD. This integrative biomarker approach may help distinguish necroptotic signaling from apoptosis-related processes and provide insight into the regional and temporal heterogeneity of inflammation-associated neuronal degeneration.

## 4. Pyroptosis in AD

Pyroptosis is a regulated inflammatory form of cell death that occurs via inflammasome-dependent activation of inflammatory caspases. Upon sensing danger-associated signals, canonical inflammasomes activate caspase-1, whereas non-canonical pathways activate caspase-4 and caspase-5 in humans. These caspases cleave gasdermin D (GSDMD) to generate an N-terminal fragment that oligomerizes within the plasma membrane, forming pores that drive cell swelling, membrane rupture, and the release of pro-inflammatory cytokines such as IL-1β and IL-18. Importantly, inflammasome assembly alone does not equate to pyroptosis; terminal execution requires gasdermin-mediated membrane permeabilization, which distinguishes inflammatory signaling from irreversible lytic destruction. In AD, pyroptosis-related signaling appears most prominent in microglia, and has also been reported in neurons and astrocytes, potentially amplifying neuroinflammation and degeneration [6].

### 4.1. Neuropathological and Experimental Evidence for Pyroptotic Neurodegeneration in AD

Evidence indicates that inflammasome activation and downstream pyroptosis-related mechanisms contribute to AD-associated neuroinflammation and neurodegeneration [9,10,67]. Analyses of postmortem human AD brains have revealed sustained inflammasome engagement in vulnerable cortical and hippocampal regions, marked by increased NOD-like receptor family pyrin domain containing 3 (NLRP3) sensor abundance, ASC adaptor accumulation, and active caspase-1 [9,68,69]. These findings indicate persistent activation of inflammasome signaling in the diseased brain rather than a transient or secondary inflammatory response. Experimental modulation of NLRP3–caspase-1 signaling in AD-relevant models alters the neuroinflammatory burden, neuronal survival, and cognition, supporting a pathogenic role for inflammasome-driven pyroptotic mechanisms in AD progression [70,71,72].

In transgenic AD mouse models, such as APP/PS1, suppression of the NLRP3–caspase-1 signaling attenuates neuroinflammation, preserves synaptic integrity, and improves cognition [71,72]. Pharmacological inhibition of NLRP3 reduces inflammatory burden and neuronal dysfunction, with modest and context-dependent effects on amyloid plaque load, indicating that inflammasome signaling promotes injury independently of large changes in Aβ [70,72]. Targeting caspase-1 similarly improves cognition and reduces inflammation-associated synaptic deterioration, further supporting a functional role for inflammasome signaling beyond a passive inflammatory response [71]. Post-symptomatic interventions have shown that inhibiting NLRP3 after pathological onset remains effective in reducing microglial activation and neurodegeneration, underscoring its relevance across disease stages [73].

Inflammasome activation has also been demonstrated in tauopathy models, in which NLRP3 activation influences both tau pathology and associated neurodegeneration, as shown in Tau22 mice [10]. In addition, post-symptomatic inhibition of NLRP3 has been shown to rescue cognitive impairment and mitigate amyloid- and tau-driven neurodegeneration in APP/PS1 mice [72]. While canonical pyroptosis is primarily mediated by GSDMD, recent studies suggest that alternative gasdermin family members, such as gasdermin E (GSDME), may also contribute to inflammatory neuronal injury under specific conditions. Unlike GSDMD, GSDME is cleaved by caspase-3 and has been reported to facilitate the transition from apoptosis to pyroptosis-like cell death, thereby linking apoptotic and inflammatory death pathways. GSDME-dependent cell death has been implicated in inflammatory damage and memory deficits in APP23/PS45 mice [74]. However, compared to GSDMD-dependent mechanisms, the role of GSDME in AD remains incompletely defined and requires further investigation. Consistent with this, GSDMD involvement in AD is supported by more robust evidence. Elevated cleaved GSDMD levels have been detected in AD brain tissue and cerebrospinal fluid, localized to activated microglia and degenerating neurons [75,76]. These findings indicate that pyroptosis involves both immune and neuronal populations and contributes to neuroinflammatory injury and neuronal dysfunction. Consistent with this mechanism, increased caspase-1-dependent cytokines, including IL-1β and IL-18, are observed in vulnerable regions, supporting ongoing pyroptotic signaling in the diseased brain [69,75].

Accumulating evidence indicates that pyroptotic neurodegeneration in AD is a multicellular process. Microglia respond robustly to fibrillar and oligomeric Aβ by activating the NLRP3 inflammasome, leading to caspase-1 activation, interleukin-1β release, and establishment of a self-amplifying inflammatory loop [9,72]. Neuronal pyroptosis can be triggered by pathological tau species through the activation of neuronal inflammasome sensors, resulting in GSDMD-dependent membrane disruption and cell death [77,78]. Consistent with these findings, hyperphosphorylated tau has been shown to activate the NLRP3 inflammasome and downstream pyroptotic signaling, including caspase-1 activation, GSDMD cleavage, and release of IL-1β and IL-18, thereby further supporting a link between tau pathology and inflammatory cell death pathways [79]. Astrocytes exposed to Aβ and inflammatory stimuli have been shown to engage inflammasome signaling and undergo pyroptotic injury, contributing to glial dysfunction and disruption of neuronal homeostasis [76]. Consistent with these findings, increased inflammasome activation and pyroptosis-related markers have been reported in association with disease progression in patients with mild cognitive impairment and AD [80].

Taken together, findings from human neuropathology and multiple experimental models, including both amyloid-driven and tau-related systems, support pyroptosis as an active, multicellular, and inflammation-driven mechanism of neurodegeneration in AD, rather than a secondary consequence of tissue injury.

### 4.2. Biomarkers Reflecting Pyroptotic Pathways in AD

Assessing pyroptotic activity in AD requires markers that capture key steps of the pathway, including inflammatory caspase activation, GSDMD cleavage, and downstream cytokine release. Several of these readouts are detectable in biofluids, enabling evaluation in living patients rather than relying exclusively on postmortem tissue (Table 3).

Extracellular inflammasome adaptor assemblies can be quantified as markers of inflammasome activation during upstream pathway engagement. A recent single-molecule assay demonstrated that extracellular ASC specks are measurable in human serum and CSF and are elevated in AD compared with controls, supporting ASC specks as candidate biomarkers of inflammasome assembly in vivo [68].

Patient-derived immune cells provide a practical window into canonical inflammasome activation at the level of caspase signaling. In a clinical cohort spanning mild cognitive impairment and AD, peripheral blood mononuclear cells showed increased NLRP3, caspase-1, GSDMD, and IL-1β expression. Plasma and CSF IL-1β levels were elevated and correlated with cognitive scores, supporting systemic inflammasome activation that tracks the clinical phenotype [80]. These findings justify the use of paired cellular and biofluid measures, such as PBMC inflammasome components together with plasma or CSF cytokines, to strengthen the interpretability beyond single analytes.

Downstream pathway specificity improves when GSDMD-related readouts are included because GSDMD is required for pore formation and lytic inflammatory cell death. In a CSF biomarker study, patients with AD showed higher CSF GSDMD levels along with higher CSF total tau and phosphorylated tau levels, and CSF inflammatory cytokines were positively associated with GSDMD, supporting a link between the inflammasome-related inflammatory state and gasdermin-associated signature in living patients [81]. Although cytokines such as IL-1β and IL-18 alone are not pyroptosis-specific, their concurrent elevation with upstream or gasdermin-associated markers increases pathway specificity, supporting the use of multi-analyte panels over cytokines alone [68,80,81].

Collectively, a translational biomarker framework for pyroptosis in AD can combine ASC speck quantification as a marker of inflammasome assembly, PBMC NLRP3, caspase-1, and GSDMD measures as cellular evidence of pathway engagement, and CSF or plasma cytokines plus CSF GSDMD as biofluid readouts linked to disease phenotypes. This integrated approach captures pyroptosis-related inflammatory injury across stages and supports its use in patient stratification and therapy monitoring.

## 5. Ferroptosis in AD

Ferroptosis is an iron-dependent form of RCD defined by lipid peroxide accumulation within membranes due to the failure of glutathione-dependent antioxidant defenses, particularly the loss of glutathione peroxidase 4 (GPX4) activity, which normally detoxifies lipid peroxides [82]. Excess intracellular iron catalyzes the peroxidation of polyunsaturated phospholipids, collapsing membrane integrity and inducing non-apoptotic death independent of caspases. Morphologically, ferroptotic cells exhibit mitochondrial shrinkage with condensed cristae and dense membranes, rather than chromatin condensation or necrosome-mediated rupture [83]. Unlike apoptosis or necroptosis, ferroptosis is not driven by protease cascades or kinase complexes but reflects metabolic failure from iron-dependent lipid peroxidation and redox imbalance. In AD, elevated cortical iron levels and extensive oxidative membrane damage provide convergent evidence that ferroptotic stress contributes to neuronal vulnerability and degeneration [11,84].

### 5.1. Neuropathological and Experimental Evidence for Ferroptotic Neurodegeneration in AD

Ferroptosis is a regulated, non-apoptotic form of cell death characterized by iron-dependent lipid peroxidation and impairment of the glutathione–GPX4 antioxidant defense system, leading to oxidative membrane injury independent of caspase activation. Consistent with these observations, recent evidence suggests that tau pathology is linked to ferroptosis through the disruption of iron homeostasis and ferritinophagy. Specifically, tau post-translational modifications have been shown to promote ferritin degradation, leading to intracellular iron accumulation and enhanced lipid peroxidation, thereby reinforcing the association between tau pathology and ferroptotic neuronal vulnerability [85]. GPX4 insufficiency is central, as GPX4 detoxifies phospholipid hydroperoxides whose accumulation destabilizes membranes. Neuropathological and in vivo studies further support the presence of ferroptosis-permissive conditions in AD and even prodromal stages. In patients with mild cognitive impairment and AD, multimodal in vivo measurements show reduced hippocampal glutathione (GSH), a key intracellular antioxidant required for GPX4-mediated lipid peroxide detoxification, and increased iron levels. These findings indicate that diminished antioxidant capacity and increased iron burden are key biochemical drivers of ferroptotic vulnerability in vulnerable brain regions. The degree of GSH loss and iron elevation correlates with the clinical status, consistent with the progressive redox imbalance across the disease spectrum [86].

Neurons are particularly susceptible to ferroptosis due to high metabolic demand, enrichment in polyunsaturated phospholipids, and limited iron export, amplifying vulnerability in AD. Human tissue studies have confirmed iron-linked lipid peroxidation in the AD brain, coupled with reduced ferroptosis-suppressing capacity (Table 4). Postmortem analyses have shown increased oxidative damage and reduced antioxidant defenses in lipid raft fractions, compartments closely associated with amyloid processing. Complementary studies in an ApoE-driven AD mouse model demonstrated that iron chelation reduces lipid raft-associated lipid peroxidation and amyloid-related readouts, supporting iron-associated oxidative injury as mechanistically relevant rather than incidental [87].

Experimental studies in AD-relevant cellular and animal models have demonstrated that disruption of ferroptosis-suppressing mechanisms, particularly GPX4-dependent lipid peroxide detoxification and neuronal iron export, induces lipid peroxidation-driven neuronal loss and cognitive impairment, whereas ferroptosis inhibition attenuates these phenotypes, supporting the mechanistic contribution of ferroptotic stress to AD-related neurodegeneration. In a 5xFAD model, ferroptosis resistance was strengthened by the genetic overexpression of GPX4, generating 5xFAD/GPX4 mice with increased cortical GPX4 activity and improved neuronal capacity to detoxify lipid reactive oxygen species (ROS), accompanied by reduced ferroptosis-associated signatures, attenuated neurodegeneration, and improved learning and memory performance [93]. Evidence from additional AD-relevant models beyond 5xFAD further supports the involvement of ferroptosis in neurodegeneration. Ferroportin (Fpn), the only known cellular iron exporter that maintains intracellular iron homeostasis, plays a critical role in preventing iron accumulation and subsequent ferroptotic damage. Consistent with an upstream iron-handling mechanism, Fpn downregulation has been observed in human AD brains and APP/PS1 mice. Neuron-specific deletion of Fpn using an Fpn flox/NEX-Cre strategy induced hippocampal atrophy and memory deficits accompanied by canonical ferroptotic features. Conversely, pharmacological inhibition of ferroptosis or restoration of Fpn ameliorates neuronal loss and cognitive impairment in AD-related models [12,88]. In complementary in vivo studies, intranasal administration of tetrahedral framework nucleic acids (tFNAs) to APP/PS1 mice improved performance in the Morris water maze and novel object recognition tests, restored synaptic protein expression and integrity, and attenuated ferroptosis-associated biochemical changes, including reduced malondialdehyde and iron accumulation and recovery of GSH, GPX4, and SLC7A11, a key cystine transporter that supports GSH synthesis and thereby maintains GPX4-dependent antioxidant defense. These findings suggest a link between Aβ-associated ferroptotic stress and synaptic cognitive dysfunction [90].

### 5.2. Molecular and Cellular Biomarkers of Ferroptosis in AD

Accurate characterization of ferroptosis in AD requires a multiparametric biomarker framework that reflects the defining biochemical features of this pathway rather than relying on a single readout. Because ferroptosis is driven by iron dyshomeostasis, lipid peroxidation, and impaired antioxidant defense, robust detection depends on an integrated assessment across these domains [86,87].

Elevated cerebral iron and ferritin levels are foundational indicators of a ferroptosis-permissive environment. Neuropathological studies have consistently demonstrated increased ferritin expression in cortical and hippocampal regions vulnerable to degeneration, while clinical investigations have reported that elevated ferritin levels in the CSF are associated with accelerated cognitive decline and an increased risk of progression from mild cognitive impairment to AD [62,86]. Although iron accumulation alone does not constitute ferroptotic execution, it provides the redox substrate required for lipid peroxidation and therefore serves as an upstream biomarker of ferroptotic susceptibility.

Downstream iron dysregulation and lipid peroxidation provide direct biochemical evidence of ferroptotic membrane injury. Reactive aldehydic byproducts such as 4-hydroxynonenal, malondialdehyde, and F_2_-isoprostanes accumulate within neuronal membranes and form stable adducts that are detectable by immunohistochemistry or mass spectrometry. These markers show strong associations with regional neurodegeneration and disease severity in AD, supporting their relevance as execution-proximal indicators of ferroptotic damage [91,92]. Importantly, concomitant depletion of reduced GSH or a decreased GSH:GSSG ratio further reflects the erosion of antioxidant buffering capacity and impaired detoxification of lipid hydroperoxides [86,89].

At the regulatory level, dysfunction of the GPX4-centered antioxidant axis is a defining molecular signature of ferroptosis. Transcriptomic and proteomic analyses of AD brain tissue consistently reveal reduced GPX4 expression or activity together with upregulation of acyl-CoA synthetase long-chain family member 4 (ACSL4) [93,94]. Because ACSL4 promotes the incorporation of polyunsaturated fatty acids into phospholipids that are highly susceptible to peroxidation, the combined GPX4–ACSL4 profile provides a mechanistically informative indicator of ferroptotic vulnerability rather than nonspecific oxidative stress.

These phospholipids enriched in polyunsaturated fatty acids (PUFA)-enriched phospholipids serve as substrates for iron-dependent lipid peroxidation and six-transmembrane epithelial antigen of prostate 3 (STEAP3) may further enhance ferroptotic susceptibility by expanding the labile iron pool, although its role in AD remains to be clarified. At the clinical level, the translation of ferroptotic signatures is further supported by iron-sensitive neuroimaging biomarkers.

The translation of ferroptotic signatures into clinical settings is further supported by iron-sensitive neuroimaging biomarkers. Quantitative susceptibility mapping enables the noninvasive visualization of regional iron distribution in vivo and consistently demonstrates increased magnetic susceptibility in the hippocampal and cortical regions of patients with mild cognitive impairment and AD [62,95]. These imaging measures correlate with cognitive impairment and disease progression, providing spatially resolved surrogates for ferroptosis-related iron stress that complement molecular and biochemical biomarkers.

Collectively, ferroptosis in AD is most reliably captured using integrated biomarker panels that combine indices of iron metabolism, lipid peroxidation, antioxidant failure, and iron-sensitive imaging. This convergent framework allows the discrimination of ferroptotic stress from other oxidative or inflammatory processes, supports longitudinal monitoring across disease stages, and establishes ferroptosis as a clinically measurable and therapeutically actionable dimension of AD pathobiology.

## 6. Autophagy-Dependent Cell Death in AD

Autophagy-dependent cell death refers to a form of RCD in which excessive, dysregulated, or unresolved autophagic activity contributes directly to cellular death rather than serving a purely cytoprotective function. Autophagy–lysosome pathway dysfunction is integral to AD pathobiology, linking impaired intracellular proteostasis with progressive neuronal vulnerability [96]. Converging neuropathological, molecular, and experimental data indicate that disrupted autophagic flux and lysosomal insufficiency are not merely downstream consequences of amyloid or tau accumulation but actively drive disease progression. This dysfunction primarily reflects impaired autophagic clearance, leading to the accumulation of unresolved autophagic intermediates that contribute to neuronal dysfunction and cell death. From the early compensatory activation of autophagy to the subsequent failure of autophagosome maturation, lysosomal acidification, and cargo degradation, autophagy impairment integrates protein aggregation, mitochondrial stress, and the engagement of secondary death pathways. Furthermore, evidence indicates that autophagy–lysosome dysfunction is measurable across disease stages and constitutes a biologically coherent axis for mechanistic stratification and therapeutic targeting in AD.

### 6.1. Neuropathological and Experimental Evidence for Autophagy-Associated Neurodegeneration in AD

Autophagy is essential for neuronal homeostasis, particularly in long-lived, post-mitotic neurons. In AD, neuropathological and experimental evidence demonstrates profound disruption of autophagy, contributing directly to neuronal dysfunction and death, rather than serving solely as a failed protective response [97,98,99].

Early ultrastructural studies revealed that dystrophic neurites in AD brains are densely packed with autophagic vacuoles (AVs), indicating severe impairment of degradative clearance (Table 5) [100]. Subsequent analyses confirmed a striking accumulation of double-membraned autophagosomes containing heterogeneous cytoplasmic debris, particularly in the early and intermediate disease stages [99,101]. These findings indicate that autophagy is actively initiated but fails to be completed in AD neurons, resulting in the pathological buildup of undegraded autophagic intermediates. GVD bodies, which are characteristic features of vulnerable neurons in AD, are now widely interpreted as late-stage autophagic structures that accumulate when autophagosome maturation and clearance are chronically impaired [102].

Molecular analyses further support the dysregulated initiation and resolution of inflammation. Postmortem transcriptomic and proteomic studies have revealed alterations in key regulators, including components of the Beclin-1–class III PI3K complex [13,14]. Reduced Beclin-1 enhances Aβ accumulation and accelerates neurodegeneration in models, while the deficiency of cofactors such as NRBF2 disrupts flux, impairs memory, and increases the Aβ burden [13,14]. In contrast, early-stage AD brains show upregulation of select autophagy-related transcripts, suggesting initial compensatory induction in response to misfolded proteins [103]. Together, these findings support a biphasic model in which autophagy is initially activated but becomes progressively ineffective as the disease advances [3,12]. Notably, tau pathology has also been linked to autophagy dysfunction, as hyperphosphorylated tau disrupts the microtubule network required for autophagosome transport and sequesters key components of the ESCRT-III complex. These structural and molecular disruptions impair autophagic flux and lysosomal degradation, leading to the intracellular accumulation of toxic protein aggregates and increased neuronal vulnerability [111].

Lysosomal dysfunction is central to autophagy failure in AD. Efficient autophagic degradation requires proper lysosomal acidification and protease activity. In AD neurons, lysosomes show abnormal enlargement, alkalinization, and accumulation of partially digested substrates, accompanied by reduced cathepsin activity [100,104,105]. This degradative failure is frequently accompanied by defective mitophagy, characterized by the sequestration of damaged mitochondria without efficient clearance, thereby promoting oxidative stress and bioenergetic dysfunction. Mutations in presenilin-1, a familial AD gene, impair lysosomal acidification independently of γ-secretase activity, directly linking causative mutations to defective clearance [112]. These defects compromise the final degradative step, resulting in chronic accumulation of toxic cargo and neuronal vulnerability [15].

Experimental models have provided evidence that autophagic dysfunction contributes to neurodegeneration. Genetic or pharmacological disruption of autophagic flux induces neuritic dystrophy, synaptic failure, and neuronal loss [113,114], whereas restoration of lysosomal function or enhancement of autophagic clearance ameliorates Aβ and tau pathologies and improves cognitive outcomes [14]. Importantly, excessive accumulation of stalled autophagosomes can become cytotoxic and contribute to neuronal dysfunction [112]. Collectively, these findings establish autophagy dysfunction as an active driver of neuronal degeneration in AD rather than a passive consequence of protein aggregation.

### 6.2. Molecular and Cellular Biomarkers of Autophagy–Lysosome Pathway Dysfunction in AD

Characterizing autophagy dysfunction in AD requires biomarkers that capture not only initiation but, critically, autophagic flux and lysosomal degradation capacity. Because autophagy is a dynamic, multistep process, static marker levels should be interpreted in the context of autophagic flux and lysosomal degradative capacity [106,107,115].

At the tissue level, accumulation of autophagic intermediates provides evidence of impaired autophagic flux. Increased levels of lipidated LC3 (LC3-II) together with accumulation of the autophagy adaptor p62/SQSTM1 indicate that autophagosomes form but are not efficiently degraded [101,105]. Elevated p62 levels are particularly informative, as they accumulate when ubiquitinated cargo is sequestered but not cleared [101]. These patterns are consistently observed in postmortem AD brains and across tauopathies, highlighting the shared autophagic–lysosomal failure as a common neurodegenerative mechanism [97,105].

Lysosomal biomarkers refine assessment of autophagy impairment. Reduced activity or altered processing of lysosomal proteases, including cathepsins, reflects compromised degradation [15,104]. Transcription factor EB (TFEB) is a key transcriptional regulator that promotes lysosomal biogenesis and autophagy by activating genes required for lysosomal function and cellular clearance. Recent studies have shown perturbed TFEB signaling in AD, with altered activation or nuclear translocation in neurons, potentially blunting lysosomal biogenesis and autophagy gene programs, thereby compromising lysosome renewal and stress resilience [108,116].

The translation of these signatures to biofluids has advanced rapidly (Table 6). CSF analyses have demonstrated elevated p62/SQSTM1 levels in patients with AD, consistent with stalled cargo accumulation rather than enhanced initiation [117]. Additional studies have reported alterations in mitophagy- and autophagy-regulatory proteins, including PINK1, ULK1, and TFEB, in the CSF and serum across the AD continuum [109,110,118]. These changes suggest a selective vulnerability of mitochondrial quality control and lysosomal renewal pathways.

Importantly, fluid biomarkers of autophagic dysfunction provide complementary information regarding the amyloid/tau/neurodegeneration (A/T/N) framework. Whereas amyloid and tau markers capture proteinopathy burden and neurodegeneration markers reflect neuronal injury, autophagy-related biomarkers index the cell-intrinsic degradative capacity that determines neuronal resilience [107,117]. Integration of autophagy biomarkers may therefore enable the stratification of patients by autophagic status, facilitate the monitoring of therapeutic interventions targeting the autophagy–lysosome system, and identify subpopulations most likely to benefit from autophagy-modulating strategies [107,117].

## 7. Other Cell Death Pathways in AD

### 7.1. Additional RCD and Injury Mechanisms in AD

In addition to the aforementioned major RCD pathways, several additional forms of neuronal injury and specialized cell death mechanisms have been implicated in AD. Although these processes often share upstream stressors such as oxidative damage, calcium dyshomeostasis, and Aβ toxicity, they are distinguished by their molecular execution mechanisms rather than their initiating triggers. Parthanatos, a caspase-independent form of RCD driven by excessive activation of poly (ADP-ribose) polymerase-1 (PARP-1), has received increasing attention [119]. Sustained PARP-1 activation in response to DNA damage depletes NAD^+^ pools and promotes apoptosis-inducing factor (AIF) translocation from the mitochondria to the nucleus, culminating in large-scale DNA fragmentation and neuronal death [119,120]. Elevated nuclear PAR polymers and increased PARP-1 immunoreactivity are consistently detected in AD brains, often co-localizing with plaques and tangles [121,122]. Experimental studies have shown that Aβ exposure induces PARP-1 activation and neuronal death via transient receptor potential melastatin 2 (TRPM2)-mediated calcium influx and mitochondrial dysfunction [123]. Pharmacological inhibition or genetic ablation of PARP-1 confers neuroprotection in AD models, attenuating synaptic loss, microglial activation, and cognitive decline [122]. Biomarkers of parthanatos include PAR polymer accumulation, elevated PARP-1 activity, and nuclear AIF translocation [124].

Closely related is mitochondrial permeability transition (MPT)-mediated necroptosis, arising from calcium overload and oxidative stress-induced opening of the mitochondrial permeability transition pore (mPTP). Cyclophilin D (CypD), a critical regulator of mPTP opening, is upregulated in hippocampal neurons in AD and directly interacts with Aβ, sensitizing mitochondria to permeability transition [125]. The resulting mitochondrial depolarization leads to bioenergetic collapse and necroptotic neuronal death. CypD deficiency or pharmacological inhibition of mPTP confers neuroprotection in AD models [126,127]. This pathway overlaps with excitotoxic mechanisms, as Aβ-induced calcium influx via NMDA receptors or mitochondrial calcium uniporters can precipitate MPT opening and necroptosis [128,129].

Cuproptosis has recently emerged as another potentially relevant form of RCD in AD [130,131]. Mechanistically distinct from apoptosis or ferroptosis, cuproptosis is triggered by intracellular copper accumulation, which promotes the aggregation of lipoylated tricarboxylic acid cycle proteins, particularly dihydrolipoamide S-acetyltransferase (DLAT), together with the loss of iron–sulfur cluster proteins, resulting in proteotoxic stress and mitochondrial dysfunction [130,131]. Ferredoxin 1 (FDX1), a central regulator of this pathway, facilitates the reduction of Cu^2+^ to Cu^+^ and enhances susceptibility to copper-dependent toxicity [130]. In AD, copper dyshomeostasis has been linked to Aβ aggregation, increased β-secretase (BACE1) activity, tau hyperphosphorylation, and oxidative stress, whereas core cuproptosis-related genes, including FDX1, LIAS, LIPT1, DLAT, DLD, PDHA1, and PDHB, connect this pathway to mitochondrial metabolic failure [130,131,132,133]. Although direct evidence for terminal cuproptotic execution in AD remains limited, these findings suggest that copper-dependent metabolic stress may represent an additional mechanism linking metal imbalance to neuronal vulnerability [130]. From a translational perspective, cuproptosis is currently constrained by the lack of pathway-specific biomarkers and limited human validation, but it may become relevant to future biomarker panels and therapeutic strategies targeting copper homeostasis, mitochondrial metabolism, and oxidative injury [131,132].

Additional emerging forms of cell death under investigation include oxytosis (oxidative glutamate toxicity) [134,135], NETosis (neutrophil extracellular trap-associated death) [136], and phagoptosis [137], in which stressed but viable neurons are engulfed by activated microglia. Although their precise contributions remain to be defined, their involvement underscores that AD engages multiple, overlapping death and clearance pathways that act together to drive neurodegeneration.

### 7.2. Crosstalk and Integrated Regulation of Cell Death Pathways in AD

Evidence indicates that neuronal and glial loss in AD arises from the coordinated activation of multiple RCD programs rather than a single isolated pathway (Table 7) [54,138]. AD-associated stressors, including proteotoxic stress, lysosomal dysfunction, mitochondrial impairment, oxidative damage, and chronic inflammation, can simultaneously engage multiple cell death pathways, whereas the predominant mode of execution is determined by the regulatory signaling hierarchy and the magnitude of the cellular stress burden [138,139,140]. These observations support a model in which multiple RCD programs are engaged within a coordinated regulatory network, accounting for the frequent detection of overlapping cell death signatures in vulnerable brain regions [54,138].

During early or moderate stress, neurons activate adaptive responses such as autophagy and mitophagy to maintain proteostasis and organelle integrity [96,139]. However, sustained Aβ and tau accumulation progressively impairs autophagy–lysosomal function and mitochondrial quality control, leading to reactive oxygen species accumulation, bioenergetic failure, and persistence of damaged organelles [96,139]. As compensatory capacity declines, neuronal signaling shifts toward the activation of multiple death programs, allowing apoptotic, necroptotic, pyroptotic, and ferroptotic pathways to be engaged concurrently within the same pathological milieu [138,141].

Shared molecular regulators further define pathway selection and activation hierarchy. Death receptor signaling promotes apoptotic execution when caspase activity is preserved, whereas reduced caspase function permits a shift toward necroptotic signaling, with RIPK1 acting as a central regulatory node in this transition and contributing to neuronal vulnerability in AD [141]. In parallel, inflammasome signaling in microglia and other immune cells drives pyroptotic execution and cytokine release, increasing the susceptibility of neighboring neurons to receptor-mediated death under chronic inflammation [6,142]. Gasdermin family effectors are increasingly recognized as active contributors to neurodegenerative phenotypes, and experimental studies support their functional relevance in AD models [74,142]. Mechanistic crosstalk extends beyond shared upstream triggers and involves convergence at the level of intracellular stress responses. Necroptotic activation through MLKL-mediated membrane disruption can enhance ROS generation and promote lipid peroxidation, thereby lowering the threshold for ferroptotic cell death. Conversely, ferroptotic stress, driven by iron-dependent lipid peroxidation and impaired antioxidant defense, can exacerbate mitochondrial dysfunction and sensitize neurons to intrinsic apoptotic signaling [22,135,141,143]. This process may involve an increased susceptibility to pro-apoptotic Bcl-2 family signaling. Bid and t-Bid represent a potential mechanistic link, as t-Bid promotes mitochondrial outer membrane permeabilization and may facilitate the integration of oxidative membrane stress with intrinsic apoptotic activation. Although direct evidence linking this axis to ferroptosis–apoptosis coupling in AD remains limited, this pathway provides a plausible point of convergence between lipid peroxidation, mitochondrial dysfunction, and apoptotic vulnerability. Lipid peroxidation-derived reactive aldehydes, such as 4-hydroxynonenal (4-HNE), can further modulate apoptotic signaling pathways and contribute to mitochondrial dysfunction, although the precise molecular intermediates linking ferroptotic stress to mitochondrial apoptosis remain incompletely defined [92].

The engagement of RCD pathways is further regulated by molecular checkpoints that integrate upstream stress signals with intracellular signaling states [4,144]. For example, caspase-8 functions as a critical molecular switch by suppressing RIPK1-RIPK3-MLKL-mediated necroptosis when apoptotic signaling is competent [143,145,146]. Conversely, attenuated caspase activity facilitates necroptotic execution, particularly in pro-inflammatory microenvironments [53]. In parallel, RIPK1 operates as a central scaffold that coordinates pro-survival, apoptotic, and necroptotic outcomes dictated by its post-translational modifications, notably phosphorylation and ubiquitination, and the stoichiometry of its interaction partners [64,143]. Beyond death receptor signaling, metabolic and organelle-specific homeostasis further dictate pathway selection. Mitochondrial dysfunction and redox imbalance, pathognomonic features of AD, promote non-enzymatic lipid peroxidation and sensitize neurons to ferroptosis [23,86,147], while simultaneously lowering the threshold for mitochondrial outer membrane permeabilization (MOMP) and subsequent intrinsic apoptosis [24]. Similarly, lysosomal impairment and defective autophagic flux disrupt proteostasis and facilitate the transition from adaptive autophagy to autophagy-dependent cell death [96,148]. These regulatory mechanisms operate within a convergent network of upstream stressors, including metabolic failure, iron dyshomeostasis, mitochondrial dysfunction, and chronic neuroinflammation, which collectively modulate the activation thresholds of multiple RCD pathways [86,147,149]. Consequently, the relative contributions of apoptosis, necroptosis, pyroptosis, ferroptosis, and autophagy-dependent cell death are determined by cell-type-specific vulnerability, stress intensity, and disease progression stage.

Importantly, accumulating experimental evidence demonstrates that simultaneous modulation of multiple RCD pathways provides greater neuroprotective efficacy than targeting individual pathways in isolation, supporting the concept that coordinated multi-pathway regulation is a key determinant of neuronal survival [8,150,151,152]. Experimental studies in neuronal systems have demonstrated that combined inhibition of distinct regulated cell death pathways, including concurrent targeting of necroptotic and ferroptotic mechanisms, can reduce neuronal death more effectively than single-pathway interventions. Experimental studies in neuronal systems, including models of Parkinson’s disease and cerebral injury, have demonstrated that the combined inhibition of distinct regulated cell death pathways, such as the concurrent targeting of necroptotic and ferroptotic mechanisms, confers greater neuroprotection than single-pathway interventions. These findings support the concept that coordinated multi-pathway regulation represents a conserved mechanism underlying neuronal survival and may be relevant to the pathophysiology of AD [153,154,155,156]. These findings have important therapeutic implications, indicating that strategies aimed at shared upstream regulators or combinatorial pathway modulation may be more effective than approaches focused on individual cell death programs.

At a higher level of integration, accumulating evidence supports coordinated activation of multiple cell death programs through shared signaling platforms. The concept of PANoptosis describes a form of inflammatory cell death integrating pyroptotic, apoptotic, and necroptotic components within multi-protein complexes. Although initially characterized in immune cells, similar coordinated signaling involving inflammasome activation, caspase-8 regulation, and RIPK1/RIPK3 pathways may contribute to the simultaneous engagement of multiple RCD programs in neurodegenerative conditions, including AD.

Oxidative stress and iron dysregulation add to the complementary layer of integration via ferroptotic mechanisms. Iron-associated lipid peroxidation and reduced ferroptosis-suppressor capacity have been documented in AD-relevant membrane compartments, and in vivo chelation studies support a causal role for iron-driven lipid peroxidation in disease-linked injury [87]. Ferroptotic vulnerability can intensify mitochondrial dysfunction and inflammatory signaling, lowering the threshold for additional death programs to engage under persistent metabolic and inflammatory stress [87,138]. Autophagy–lysosomal impairment further amplifies this vulnerability by limiting the clearance of damaged mitochondria and oxidized cellular constituents, accelerating the commitment toward irreversible degeneration once oxidative and inflammatory stress surpasses the compensatory capacity [96,139].

Experimental and translational studies support a staged, integrated view of RCD regulation in AD. Early phases often feature activation of autophagy-related stress responses, whereas later phases show concurrent apoptotic markers together with necroptotic, pyroptotic, and ferroptotic signatures, consistent with the progressive failure of homeostatic mechanisms and increasing dominance of execution programs [54,87,139,141]. Postmortem analyses have confirmed that end-stage AD brains exhibit heterogeneous neuronal and glial death phenotypes, with apoptotic and non-apoptotic forms coexisting across vulnerable regions [157]. This heterogeneity is further supported by single-nucleus and spatial transcriptomic studies of, sporadic early-onset AD, which reveal distinct stress-responsive and degeneration-associated programs across cell types and brain regions [158,159]. Cross-species integrative analyses have confirmed that these diverse, yet interconnected programs are conserved between human and experimental models [160].

Collectively, these findings support a model in which AD progression is driven by the integrated engagement of multiple RCD pathways rather than a single dominant mechanism, providing a mechanistic basis for combinatorial and upstream-targeted therapeutic strategies [157,158,159,161].

## 8. Methodological Challenges and Future Perspectives in RCD in AD

Although substantial progress has been made in delineating RCD mechanisms in AD, important methodological and translational challenges remain, necessitating more integrative and clinically relevant approaches to improve mechanistic interpretation and therapeutic translation. One of the central methodological constraints in interpreting RCD in AD lies in the intrinsic biological overlap among distinct cell death pathways, which are frequently co-activated under shared pathological stress conditions such as Aβ accumulation, tau pathology, oxidative stress, and chronic neuroinflammation [150,151,152]. Inflammatory cytokines such as IL-1β are closely associated with inflammasome activation and pyroptotic signaling but also represent broader neuroinflammatory responses. Likewise, oxidative stress-related markers, including lipid peroxidation products, indicate redox imbalance but are not exclusive to ferroptotic cell death. DNA fragmentation and caspase activation further exemplify this overlap, as they can occur across multiple cell death modalities or reflect secondary processes. These considerations underscore the need for integrative and multi-dimensional analytical strategies that incorporate upstream regulators, execution-phase markers, and biofluid-derived signals. Therefore, future biomarker development should therefore prioritize composite and pathway-resolved panels capable of capturing biologically meaningful distinctions within an interconnected cell death landscape.

The clinical applicability of RCD is constrained by the limited detectability of certain RCD processes. Biomarkers associated with apoptosis and inflammation are relatively accessible in biofluids, whereas ferroptosis and autophagy-related processes remain difficult to assess in patients. Ferroptosis is typically inferred from indirect indicators such as iron accumulation and lipid peroxidation, whereas autophagy-related markers frequently reflect impaired flux rather than active cell death. Therefore the development of pathway-resolved, in vivo-applicable biomarkers represents a critical priority for future research. The ability to capture neurons undergoing active cell death also remains restricted, as postmortem analyses largely reflect end-stage pathology and provide indirect evidence of preceding events. Experimental systems may also introduce additional constraints, as the transgenic models reproduces the key features of Aβ and tau pathology, may not fully reflect the extent or temporal dynamics of neuronal loss observed in human disease. A substantial proportion of mechanistic evidence is derived from familial AD models, which do not fully represent the multifactorial nature of sporadic AD, including aging, metabolic dysregulation, vascular contributions, and chronic systemic inflammation. Future research should prioritize the development of models that incorporate aging-related and metabolic contexts to improve translational relevance. In addition, inter-individual heterogeneity adds an additional layer of complexity, as genetic background, metabolic status, and immune profiles may influence the relative contributions of distinct cell death pathways across patient subgroups. A further limitation lies in the incomplete characterization of the temporal dynamics and hierarchical relationships among RCD pathways. Future studies integrating longitudinal designs, single-cell and spatial omics, and advanced in vivo imaging will be essential to define when and where distinct RCD programs predominate and to identify critical windows for therapeutic intervention. Importantly, emerging evidence suggests that metabolic state is a central determinant of neuronal vulnerability and cell death pathway engagement in AD. Alterations in energy metabolism, mitochondrial function, and redox homeostasis are well-established features of AD and contribute to bioenergetic failure and oxidative stress in vulnerable neurons [147]. Recent studies have further demonstrated that distinct metabolic signatures are associated with differential neuronal vulnerability and disease progression, supporting the role of metabolic remodeling as a key regulator of heterogeneous cell death outcomes in AD [157].

Emerging evidence further suggests that metabolic instability, including glycemic variability, represents a systemic driver of neurodegenerative processes across multiple disorders, through its contribution to oxidative stress, mitochondrial dysfunction, and neuroinflammatory activation [22,138,147]. In particular, Parkinsonian disorders have been increasingly associated with fluctuations in glucose homeostasis, which contribute to oxidative stress, mitochondrial dysfunction, and neuroinflammatory activation. These processes converge on shared cellular vulnerability pathways and may influence the engagement of regulated cell death mechanisms [160,162]. Within this framework, metabolic dysregulation may act as a cross-disease modifier of neuronal vulnerability, supporting the applicability of regulated cell death frameworks across neurodegenerative diseases, including AD and Parkinsonian disorders.

Collectively, these challenges support a conceptual shift from single-pathway interpretations toward integrated, network-based models of neurodegeneration. Therefore, therapeutic strategies are therefore expected to evolve beyond isolated pathway inhibition toward combinatorial or upstream-targeted approaches that address shared regulatory mechanisms, including metabolic dysfunction, redox imbalance, and inflammatory signaling.

## 9. Conclusions

Neuronal loss in AD results from the coordinated engagement of multiple RCD pathways rather than a single dominant mechanism. Apoptosis, necroptosis, pyroptosis, ferroptosis, and autophagy-associated cell death are mechanistically interconnected and variably activated across cell types and disease stages. This complexity indicates that targeting individual death pathways in isolation is unlikely to provide durable neuroprotection. Collectively, these observations support the concept that therapeutic strategies targeting upstream regulators or integrating multiple pathways may offer greater efficacy than approaches focused on a single cell death mechanism, given the context-dependent and interconnected nature of RCD in AD. Instead, therapeutic and biomarker strategies that address shared upstream stressors and capture pathway-specific activity will be essential for preserving neuronal vulnerability and modifying disease progression.

## Figures and Tables

**Table 1:** Molecular and cellular biomarkers of apoptotic signaling in AD.

Apoptotic Signaling	Representative Markers	Model Evidence [Refs]	Significance in AD
Nuclear late stage execution	TUNEL, DNA fragmentation	Human AD cortex /hippocampus [19,20]	Terminal DNA cleavage in plaque- and tangle-associated vulnerable neurons
Extrinsic and intrinsic initiation	Caspase-8, Caspase-9	Aβ- and inflammation-induced models [30,32,34]	Engagement of death receptor and mitochondrial apoptotic cascades under Aβ and inflammatory stress
Terminal execution	Cleaved caspase-3	AD neurons and 5xFAD brain [37,41]	Active execution-phase proteolysis associated with synaptic and neuronal loss
Mitochondrial intrinsic pathway	Bax/Bcl-2 ratio, BAD	5xFAD mouse models [38,39,40,42]	Pro-apoptotic Bcl-2 family imbalance promoting mitochondrial outer membrane permeabilization
Caspase-independent mitochondrial signaling	AIF nuclear translocation	Human AD hippocampus [43]	Mitochondria-derived caspase -independent neuronal death mechanism
Stress-activated regulatory signaling	c-Jun, c-Fos	AD-associated stress [44]	MAPK/JNK-driven transcriptional activation priming neurons for apoptotic vulnerability

Abbreviations: AD, Alzheimer’s disease; AIF, apoptosis-inducing factor; BAX, Bcl-2-associated X protein; Bcl-2, B-cell lymphoma 2; MAPK, mitogen-activated protein kinase.

**Table 2:** Canonical necroptotic signaling biomarkers in Alzheimer’s disease reference.

Biomarker Category	Representative Markers	Evidence [Refs]	Significance in AD
Molecular signaling axis	RIPK1, RIPK3	Human AD hippocampus and cortex [54,57]	Increased RIPK1/RIPK3 expression and activation in disease-vulnerable regions, associated with tau pathology and neuroinflammatory signaling
Execution marker	Phosphorylated MLKL	Human AD tissue; enrichment in GVD lesions [58,59,60]	p-MLKL accumulation in vulnerable neurons, particularly within GVD structures, correlates with Braak stage and neuronal loss
Histopathological correlate	p-MLKL–positive GVD neurons	Human hippocampus; correlation with Braak stage [59,60]	Spatial association of necroptotic activation with tau-associated neurodegeneration and region-specific neuronal vulnerability
Regulatory interaction	Reduced caspase-8 activity with elevated RIPK3/p-MLKL	Human AD tissue [60,61]	Impaired caspase-8-mediated regulation of RIPK1/RIPK3 signaling
Translational imaging biomarker	RIPK1-targeted PET tracers	In vivo AD models [57,62]	PET radioligands targeting RIPK1 ([^11^C]7k, [^18^F]7i, and [^18^F]8a) demonstrate brain penetration and elevated RIPK1 signal in AD models.

Abbreviations: AD, Alzheimer’s disease; GVD, granulovacuolar degeneration; MLKL, mixed lineage kinase domain-like protein; p-MLKL, phosphorylated MLKL; RIPK1, receptor-interacting serine/threonine-protein kinase 1; RIPK3, receptor-interacting serine/threonine-protein kinase 3.

**Table 3:** Inflammasome-associated pyroptotic biomarkers in Alzheimer’s disease.

Pathway Level	Biomarkers	Evidence [Refs]	Relevance in AD
Inflammasome assembly	NLRP3, ASC specks	Human AD brain; serum/CSF ASC assays [10,67]	Sustained inflammasome activation in vulnerable cortical and hippocampal regions consistent with chronic neuroinflammation
Inflammatory caspase activation	Caspase-1	Human AD tissue; AD mouse models [68,69,70]	Canonical inflammasome signaling linked to microglial activation and neuronal dysfunction
Execution-phase pore formation	Cleaved GSDMD	Human AD brain and CSF [72,73,78]	Systemic NLRP3–caspase-1–GSDMD activation associated with clinical progression from amnestic MCI to AD
Cytokine release	IL-1β, IL-18	Human tissue and CSF/plasma [67,77]	Amplification of neuroinflammatory cascades associated with synaptic impairment and cognitive decline
Peripheral cellular markers	PBMC NLRP3, caspase-1, GSDMD	Clinical cohort studies [77]	Systemic inflammasome activation associated with central inflammatory pathology in AD

Abbreviations: AD, Alzheimer’s disease; ASC, apoptosis-associated speck-like protein containing a CARD; CSF, cerebrospinal fluid; GSDMD, gasdermin D; IL-1β, interleukin-1 beta; IL-18, interleukin-18; NLRP3, NOD-like receptor family pyrin domain containing 3; PBMC, peripheral blood mononuclear cell.

**Table 4:** Ferroptosis-related molecular and translational signatures in Alzheimer’s disease.

Mechanistic Level	Representative Markers	Evidence [Refs]	Pathophysiological Relevance in AD
Iron accumulation	Elevated cortical iron; ferritin increase; ferroportin reduction	[11,87,88,89]	Regional iron retention associated with lipid peroxidation, synaptic dysfunction, and cognitive impairment in AD
Lipid peroxidation execution	4-HNE; MDA; F_2_-isoprostanes	[62,90]	Oxidative modification of neuronal membrane lipids linked to neurodegeneration and disease severity
Antioxidant defense impairment	Reduced GSH; altered GSH:GSSG ratio	[11,91]	Impaired detoxification of lipid peroxides contributing to neuronal vulnerability
GPX4-centered regulatory control	Decreased GPX4; ACSL4 upregulation	[86,92]	Altered ferroptosis regulatory balance promoting membrane phospholipid peroxidation in AD models
Causal modulation in vivo	GPX4 overexpression; ferroptosis inhibition	[12,86,87,93]	Neuroprotection and cognitive improvement following ferroptosis suppression in AD mouse models

Abbreviations: AD, Alzheimer’s disease; ACSL4, acyl-CoA synthetase long-chain family member 4; GSH, reduced glutathione; GPX4, glutathione peroxidase 4; GSSG, oxidized glutathione; MDA, malondialdehyde.

**Table 5:** Cellular autophagy–lysosome pathway dysfunction in Alzheimer’s disease.

Autophagy Process	Representative Markers	Evidence [Refs]	Contribution to Neurodegeneration in AD
Autophagy initiation	Reduced Beclin-1; altered autophagy transcripts	Human AD brain; models [13,101,102]	Beclin-1 deficiency associated with increased Aβ accumulation and enhanced neurodegenerative vulnerability
Autophagic flux impairment	Increased LC3-II, p62/SQSTM1; autophagic vacuole accumulation	EM and postmortem AD brain [97,100,101]	Autophagosome accumulation with incomplete lysosomal clearance contributing to neuritic dystrophy and synaptic dysfunction
Lysosomal degradation failure	Cathepsin dysfunction; lysosomal alkalinization; PS1-related defects	Human tissue and PS1 models [14,103,104,105]	Defective autolysosomal acidification and proteolysis linked to intracellular Aβ retention and progressive neuronal injury
Lysosomal biogenesis regulation	Impaired TFEB activation/ nuclear translocation	Human and mouse studies [106,107]	Reduced lysosomal biogenesis associated with impaired proteostasis and intracellular aggregate accumulation in AD neurons
Mitophagy-associated alterations	PINK1, ULK1 dysregulation	Clinical and experimental data [108]	Impaired mitochondrial quality control associated with increased reactive oxygen species accumulation and bioenergetic dysfunction
Crosstalk with apoptosis	Caspase-cleaved Beclin-1; BAX/p53 modulation	Experimental studies [109,110]	Autophagy–apoptosis interface facilitating transition from degradative stress response to apoptotic signaling in AD

Abbreviations: AD, Alzheimer’s disease; LC3, microtubule-associated protein 1 light chain 3; PINK1, PTEN-induced kinase 1; PS1, presenilin-1; SQSTM1, sequestosome 1; TFEB, transcription factor EB; ULK1, Unc-51-like kinase 1.

**Table 6:** Translational fluid biomarkers of autophagy–lysosome dysfunction in Alzheimer’s disease.

Biomarker Type	Biomarkers	Evidence [Refs]	Clinical Relevance
Autophagy cargo accumulation	CSF p62/SQSTM1	Clinical AD cohorts [117]	Impaired autophagic cargo clearance associated with AD pathology
Mitophagy-related proteins	PINK1, ULK1	CSF and serum studies [108,118]	Altered mitochondrial quality control linked to neuronal vulnerability
Lysosomal biogenesis regulation	TFEB-related alterations	Human biofluid analyses [108,116]	Reduced lysosomal biogenesis capacity associated with autophagy–lysosome dysfunction
Therapeutic modulation of autophagy– lysosome signaling	TFEB activation/ acetylation	AD mouse models [108,116,117]	Lysosomal biogenesis activation associated with reduction in AD-related pathological phenotypes

Abbreviations: AD, Alzheimer’s disease.

**Table 7:** Integrated regulatory architecture of RCD in Alzheimer’s disease.

Regulatory Axis	Core Mechanistic Determinant	Relevance to AD Pathobiology
Shared upstream stress network	Proteotoxic burden (Aβ, tau), oxidative stress, lysosomal dysfunction, Ca^2+^ imbalance	Establishes a multi-permissive environment enabling co-activation of apoptotic and non-apoptotic RCD programs
Pathway selection control	Caspase activity, RIPK1/RIPK3 signaling, inflammasome status, GPX4-dependent redox buffering	Determines relative predominance of apoptosis, necroptosis, pyroptosis, or ferroptosis in vulnerable regions
Mitochondrial-metabolic integration	mPTP activation, NAD^+^ depletion, lipid peroxidation	Couples bioenergetic collapse to irreversible neuronal degeneration
Temporal disease integration	Progressive decline in adaptive capacity (autophagy, redox control)	Transition from compensatory stress responses to simultaneous multi-pathway RCD activation in advanced AD

Abbreviations: AD, Alzheimer’s disease; Aβ, amyloid beta; Ca^2+^, calcium ion; GPX4, glutathione peroxidase 4; mPTP, mitochondrial permeability transition pore; NAD^+^, nicotinamide adenine dinucleotide; RIPK1, receptor-interacting serine/threonine-protein kinase 1; RIPK3, receptor-interacting serine/threonine-protein kinase 3; RCD, regulated cell death.

## Data Availability

No new data were created or analyzed in this study. Data sharing is not applicable to this article.
